# Cadmium(II) inhibition of human uracil-DNA glycosylase by catalytic water supplantation

**DOI:** 10.1038/srep39137

**Published:** 2016-12-15

**Authors:** Trevor Gokey, Bo Hang, Anton B. Guliaev

**Affiliations:** 1 Department of Chemistry and Biochemistry, San Francisco State University, 1600 Holloway Ave, San Francisco, CA 94132, USA; 2 Biological Systems and Engineering, Lawrence Berkeley National Laboratory, 1 Cyclotron Road, Berkeley, CA 94720, USA

## Abstract

Toxic metals are known to inhibit DNA repair but the underlying mechanisms of inhibition are still not fully understood. DNA repair enzymes such as human uracil-DNA glycosylase (hUNG) perform the initial step in the base excision repair (BER) pathway. In this work, we showed that cadmium [Cd(II)], a known human carcinogen, inhibited all activity of hUNG at 100 μM. Computational analyses based on 2 μs equilibrium, 1.6 μs steered molecular dynamics (SMD), and QM/MM MD determined that Cd(II) ions entered the enzyme active site and formed close contacts with both D145 and H148, effectively replacing the catalytic water normally found in this position. Geometry refinement by density functional theory (DFT) calculations showed that Cd(II) formed a tetrahedral structure with D145, P146, H148, and one water molecule. This work for the first time reports Cd(II) inhibition of hUNG which was due to replacement of the catalytic water by binding the active site D145 and H148 residues. Comparison of the proposed metal binding site to existing structural data showed that D145:H148 followed a general metal binding motif favored by Cd(II). The identified motif offered structural insights into metal inhibition of other DNA repair enzymes and glycosylases.

Many metals are ubiquitous in the environment and have many uses in both industry and daily life. The general population is exposed to metals through occupational exposure, food, water, air, and a variety of consumer products. Metal ions such as cadmium [Cd(II)] can accumulate in the body causing an age-dependent increase in internal levels. Both the IARC (International Agency for Research on Cancer)^1^ and NTP (National Toxicology Program)^2^ classified cadmium as a human carcinogen and is a well-known human lung and prostate carcinogen^3^,^4^, which are the two most prevalent and lethal cancers for men in the United States^5^. For cigarette smokers cadmium concentrations in lung tissues can be 2–4 fold higher than those of non-smokers^6^. The mechanisms used by metal ions which promote malignancy are still not fully understood. Among several possible mechanisms proposed, including genotoxicity and epigenetic effects, metal ions have been shown to inhibit DNA repair and several repair enzymes or steps in specific repair pathways^2^,^7^,^8^,^9^,^10^,^11^,^12^,^13^,^14^,^15^. These data suggest that inhibition of DNA repair may be an important contributing factor in cancer etiology. Deficiency in repair pathways, either inherited or acquired, can lead to increased risk of genomic instability that may lead to cancer^3^,^16^,^17^.

We have previously demonstrated that Cd(II), Ni(II), Pb(II), and Zn(II) have moderate to strong inhibitory effects on methylpurine-DNA glycosylase (MPG, also known as AAG or APNG)^18^. MPG, like other glycosylases, is responsible for the initial detection and excision of toxic/mutagenic alkylated bases in DNA in the base excision repair (BER) pathway. Alkylated bases are commonly occurring and highly mutagenic lesions that arise from both endogenous and exogenous sources^19^. Uracil is another common lesion found in DNA and is caused by normally occurring spontaneous C→U deamination and is a major pro-mutagenic event causing G:C→A:T transition mutations if left unrepaired. Uracil-DNA glycosylase (UNG) is the primary BER enzyme which excises uracil from DNA and is one of the most specific and efficient glycosylases in the BER pathway^20^. UNG also recognizes variety of other lesions^21^,^22^,^23^, and has been shown to prevent viral integration of HIV1 in human cells^24^.

UNG is functionally similar to MPG and utilizes a base flipping mechanism to excise lesions^3^. UNG, like MPG, does not require metal for catalytic activity and belongs to a large group of DNA repair enzymes that do not utilize metals for function and/or structure. This group of BER enzymes includes AlkA (*E. coli* analog of MPG), thymine-DNA glycosylase (TDG) and its *E. coli* analog, MUG, and also single-strand selective UNG (SMUG1) and NEIL1^25^. In contrast to the above glycosylases, divalent metals play an essential role in the catalytic reactions of the other BER enzymes. These enzymes include apurinic/apyrimidinic endonuclease (APE1) and the A/G-mismatch-specific adenine glycosylases MutY/Mig. It was proposed that the inhibitory effect of divalent metals on APE1 may be associated with their specific interaction with the active site residues^26^. The Mg(II)-dependent catalysis of APE1 can be disrupted by inhibitory metals occupying the Mg(II) binding sites but with different coordination chemistry or ligand preferences. UNG, being a non-metal requiring glycosylase, utilizes several structural features to excise lesions. Unique to UNG is a segment called the leucine probe loop (268-HPSPLSVYR-276) which stabilizes the binding of damaged DNA by inserting L272 into the space previously occupied by the flipped out lesion^27^. The lesion fills the binding pocket of UNG which is highly specific to uracil and similar derivatives. With the damaged base flipped, the DNA backbone ribose is exposed and attacked by a nucleophilic water molecule activated by the highly conserved water-activating loop (145-DPYH-148) of UNG. Glycosidic cleavage produces 5-hydroxy ribose and an apurinic (AP) site in DNA. Generation of such AP sites initiates the BER pathway, effectively making glycosylases the key to BER. Inhibition of glycosylases such as UNG would cause both exogenous (e.g. oxidative stress) and endogenous (e.g. spontaneous decay) DNA damage to remain undetected. Determining the ability of metal ions to inhibit glycosylases is therefore critical for understanding mechanisms responsible for DNA repair deficiency.

In this work we tested the ability of Cd(II) ions to inhibit human UNG (hUNG) at μM concentrations. Although the work by us and others clearly demonstrated that toxic metals inhibit DNA repair, there is no structural data available for specific details on protein-metal interactions. To address this, computational methods in this work investigated the structural details and interactions between toxic metal and a non-metal requiring DNA repair protein such as UNG.

## Results

### Assays

Previous biochemical studies have identified several repair proteins that are targets for specific metal ions and their mechanisms of interactions proposed. We also previously showed that the activity of MPG, a BER enzyme that does not apparently require a metal for its function or structure, is inhibited by metal ions including Cd(II)^18^.

**Figure 1:**
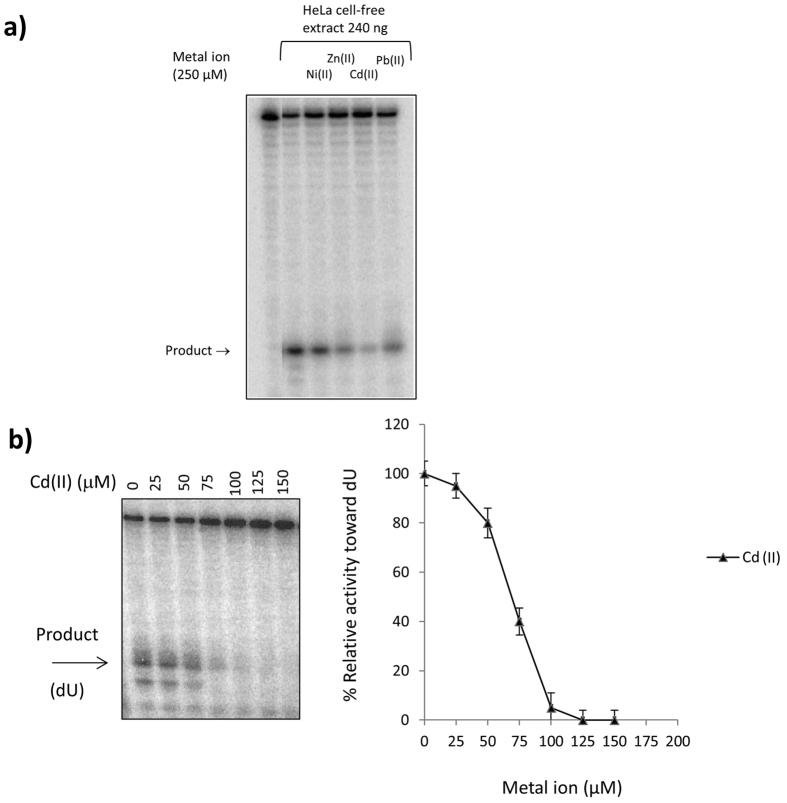
(**a**) Metal inhibition of U:G-DNA glycosylase activity in HeLa cell-free extracts. Note the strong inhibition by Cd(II) in cell-free extracts. (**b**) Cleavage assay (left panel) of recombinant hUNG against a 25-mer oligonucleotide containing dU under various concentrations of metal ion. Enzymatic reactions were carried out at 37 °C for 30 min as described in the Methods. All reactions were terminated by addition 5 μL of an alkaline solution (NaOH/formamide) to 10 μL of the reaction mixture followed by heating samples at 100 °C for 3 min to cleave the AP sites generated by hUNG. The arrow indicates the 5-mer marker position which is the expected position for a cleavage product. The right panel shows relative cleavage activity of hUNG toward dU under various concentrations of metal cations. Band areas on the phosphorimage were measured for quantitation corresponding to the cleaved product and the remaining uncut substrate. The activity of UNG for dU without the presence of metal ions was treated as 100%. The data were an average value of three parallel experiments.

In this work, inhibition of UNG activity was observed when cell-free extracts of HeLa cells were tested for activity against dU in the presence of 4 metals [Cd(II), Zn(II), Ni(II), or Pb(II)] where Cd(II) appeared to be the most prominent inhibitor (Fig. 1a). However, the dU excision activity measured in HeLa cell-free extracts reflected the activity of several enzymes. To investigate possible inhibition of hUNG by Cd(II), the enzyme mainly responsible for dU activity, we tested the activity of a recombinant hUNG towards an oligonucleotide containing dU. The glycosylase activity was tested under various concentrations of the metal at 37 °C for 30 min followed by addition of an alkaline solution containing NaOH/formamide to cleave the resulting AP site. Separation of intact oligonucleotide (25-mer) and cleaved product (5-mer) was achieved with denaturing PAGE (details in the Methods). As shown in Fig. 1b, hUNG was rendered inactive in the presence of Cd(II) at 100 μM and above as shown by the lack of the intensity of the expected 5-mer cleavage product. At 75 μM Cd(II) concentration the activity of the enzyme was reduced to 40%. To investigate the mechanism of UNG inhibition by Cd(II) we turned to the computer simulations of UNG surrounded by Cd(II) ions.

### Cd(II) binding of the hUNG active site

**Figure 2:**
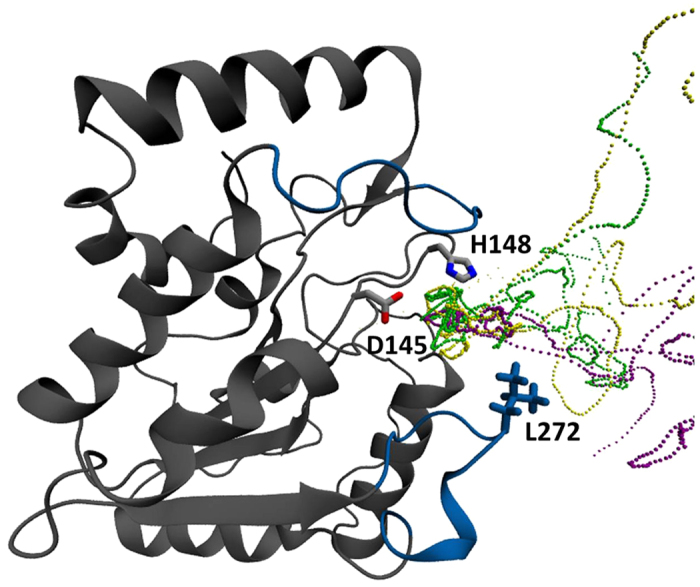
Molecular dynamics trajectory of Cd(II) attraction from bulk solvent into the immediate proximity of D145. The three different colors represent trajectory traces of Cd(II) paths entering the binding groove over the course of 1 μs of MD simulation in explicit solvent.

**Figure 3:**
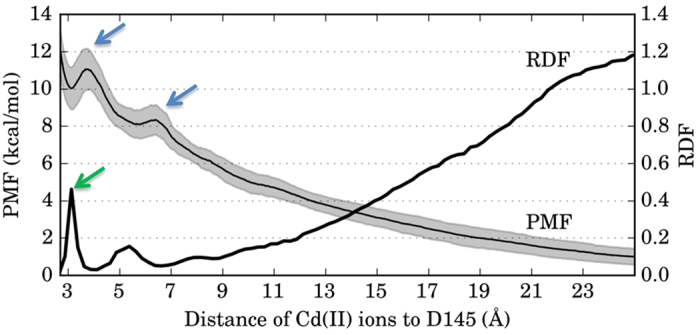
Work (PMF) needed to pull a single Cd(II) ion into the hUNG active site from SMD simulations overlaid with the radial distribution function (RDF) of Cd(II) ions in separate non-pulling equilibrium MD simulations. Work was calculated based on 1.6 μs of SMD simulations (4 repeats of 400 ns each; standard deviation is shown in grey). Two barriers (6.5 Å and 3.8 Å away from D145) were clearly observed during the SMD simulations (labeled by blue arrows). The first barrier corresponded to the initial entrance of Cd(II) into the DNA binding groove and the second corresponded to Cd(II) entering the binding groove with rearrangement of several proximal residues (see text). There was a strong correlation between the RDF of Cd(II) ions around D145 and the PMF calculated from the SMD simulations (*r*^2^ = 0.9025). The concentration peak around 3 Å (green arrow) corresponded to the accumulation of the Cd(II) near D145.

Similar to our previous work^18^ we employed Molecular Dynamics (MD) simulations to study Cd(II) interaction with hUNG. The simulation showed various areas of Cd(II) localization around the negatively charged solvent-exposed residues with some ions clearly entering the DNA binding groove and interacting with catalytic D145 (Fig. 2). Visual analysis of the MD trajectory showed that Cd(II) localization around D145 was isolated from bulk solvent and the rest of the DNA binding groove showed reduced occupancy. This was further supported by the radial distribution function (RDF) calculations shown in Fig. 3, which showed two distinct localizations of Cd(II) near D145. The major Cd(II) concentration peak was observed 3 Å away from D145 (labeled by a green arrow) and identified the preferred ion location in the active site. This indicated that Cd(II) ions were required to pass an energy barrier to reach D145. We investigated this barrier using steered molecular dynamics (SMD), where a single Cd(II) ion was pulled towards D145 from bulk solvent. Two barriers were revealed using four independent SMD simulations with a pulling velocity of 0.075 Å/ns. The first barrier appeared when Cd(II) was 6.5 Å away from D145 and corresponded to the initial entrance of Cd(II) into the DNA binding groove (Fig. 3). The second barrier appeared when Cd(II) was 3.8 Å away from D145 and corresponded to Cd(II) overcoming steric clash with surrounding residues, specifically N144, S270, and A214 appeared to be the closest (3 Å) to Cd(II). In addition to characterizing the two energy barriers using a single Cd(II) ion, the SMD simulations were also used to assess any effects caused by a high number of ions in the equilibrium simulation. If no such effects were present, the RDF (50 ions) and PMF (1 ion) should be nearly identical. The character of the RDF was in agreement with the PMF calculated from the SMD simulations with a squared correlation coefficient of *r*^2^ = 0.9025. Since both simulation techniques found identical Cd(II) localizations near D145, we confirmed that the high Cd(II) concentration in the MD simulations did not introduce unphysical behavior. This eluded that the dynamics of hUNG in the equilibrium MD and non-equilibrium SMD simulations were consistent and in good agreement.

**Figure 4:**
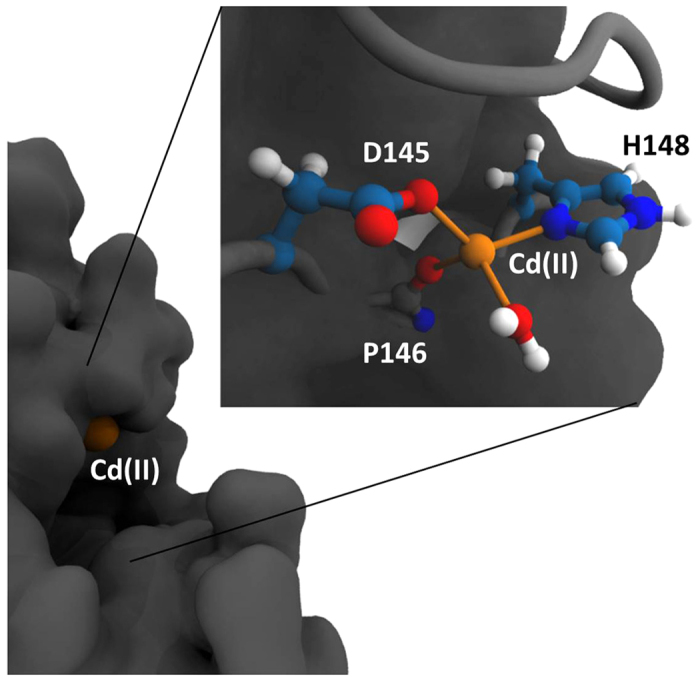
The Cd(II) coordination site produced by DFT geometry refinement using the ONIOM method. The metal (shown in orange) bound in a tetrahedral coordination utilizing D145, P146, and H148 residues of the hUNG active site and one nearby water molecule. The active site residues and coordination water molecule are shown as individual atoms.

To investigate whether Cd(II) interaction was purely electrostatic or if a stable coordination site could form with D145 and adjacent H148 or H268 residues, we continued the simulations using QM/MM MD with the PM3 model on the Cd(II) ion, and the D145, P146, H148, and H268 residues. After 4 ns of simulation time the metal ion moved in between D145 and H148 revealing a possible binding site with these two residues. In this position the Cd(II) ion occupied the same location as the catalytic water found in multiple crystallized structures with or without bound DNA (PDB ID 1AKZ, 1SSP). We then refined the geometry of this position using DFT with the ONIOM method in Gaussian09. The resulting geometry was a nearly regular tetrahedron utilizing D145, H148, P146, and one water molecule (Fig. 4) and was found to have no imaginary frequencies indicating that the geometry was stable. The distance from the metal ion to the carboxylate of D145 was 2.14 Å, the H148 N_δ_ was 2.21 Å, the P146 amide oxygen was 2.22 Å, and the water oxygen was 2.26 Å.

### hUNG dynamics of Cd(II) binding

**Figure 5:**
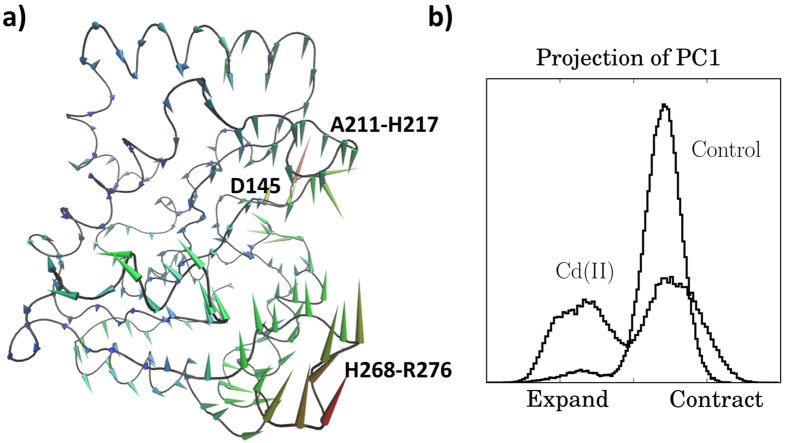
(**a**) The average structure of hUNG from MD simulations with Cd(II) ions showing the motion described by the first principal component (PC1). The cones point in the direction corresponding to positive displacement along PC1. The cone lengths show the relative contribution to the major motion. Positive (negative) displacements along PC1 indicated that the DNA binding groove contracted (expanded). The area with the largest displacement corresponded to the leucine probe loop (H268-R276). (**b**) PC1 displacement of the MD simulations with Cd(II) ions and without ions (control). PC1 of the control simulation was Gaussian and indicated a conserved protein conformation. PC1 of the Cd(II) simulation showed two peaks indicating an anharmonic shift in the conserved conformation. The observation of the two peaks in the Cd(II) simulation represented two distinct conformations corresponding to expansion and contraction of the two interfacing loops of the DNA binding groove.

We further analyzed the MD simulations to determine how the conformational dynamics of hUNG responded to Cd(II) ions entering the DNA binding groove. Principle component analysis (PCA) was used to compare the dynamics of hUNG in the presence of Cd(II) ions using a 1 μs metal-free simulation as a control. From the 645 components calculated from 215 alpha carbons of hUNG, the first 13 components were sufficient to describe 90% of hUNG motion. Figure 5a shows the major motion of hUNG during the simulations as described by the first principle component (PC1). PC1 of the control simulation was Gaussian, i.e. harmonic, but PC1 of the Cd(II) simulation showed two peaks, i.e. anharmonic (Fig. 5b). All other components in both simulations were Gaussian and thus described harmonic motions. The observation of the two peaks in the metal simulation corresponded to the expansion and contraction of two loops of the DNA binding groove. Expansion corresponded to the leucine probe loop (268-HPSPLSVYR-276) and the opposing A211-H217 loop moving away from each other while contraction brought them closer together. Binding of DNA by hUNG is likely enhanced by the flexibility of these two loops since PC1 represented a motion orthogonal to the principle axis of bound DNA, and it is possible to suggest that PC1 described a motion resembling L272 insertion into bound DNA.

**Figure 6:**
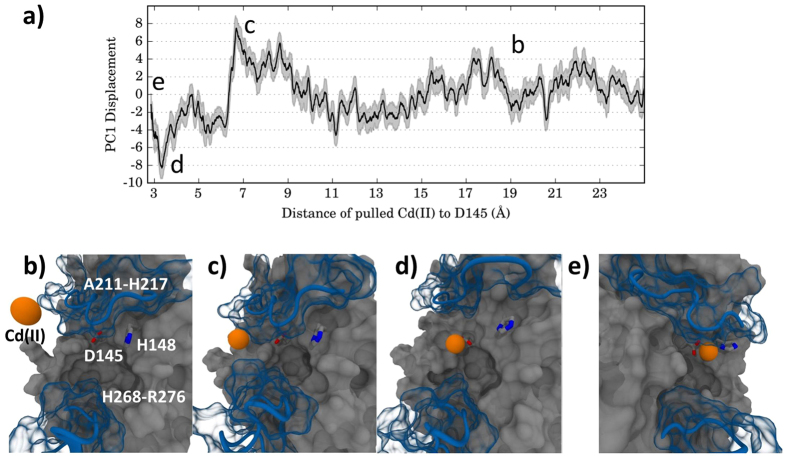
(**a**) The PC1 displacement as a function of Cd(II) distance to the active site D145 residue based on SMD simulations. Negative displacements corresponded to expansion where the leucine probe loop (268-HPSPLSVYR-276) and the opposing A211-H217 loop moved away from each other while positive displacements corresponded to a contraction bringing the two loops closer together. **(b–e)** Representative conformations showing hUNG response to Cd(II) approaching the active site. Panel b shows hUNG in its native conformation without interaction with Cd(II). Panels c, d, and e correspond to the contraction, expansion, and bound conformation of hUNG with Cd(II), respectively. Cd(II) is displayed in orange and the DNA binding groove loops in blue.

The projection of the SMD trajectories onto PC1 (Fig. 6a) showed that the expansion and contraction of the binding groove was harmonic when the Cd(II) ion was at non-interacting distances with the hUNG active site (Fig. 6b). As the ion approached interacting distances, PC1 displacement increased and indicated that hUNG’s first response was to contract the DNA groove and this contraction was maximum when Cd(II) was directly adjacent to the groove (Fig. 6c). Cd(II) entrance into the groove caused an immediate expansion (Fig. 6d) shown by a sharp decrease in PC1 displacement around 6 Å (Fig. 6a) which also corresponded to the first energy barrier in the SMD PMF (Fig. 3). This expanded state was largely unobserved in the metal-free control simulation. Further Cd(II) passage towards D145 required overcoming the second energy barrier which was determined to be additional expansion of the DNA binding groove and rearrangement of proximal residues. hUNG continued to expand as Cd(II) moved deeper into the groove and maximum expansion occurred at the observed energy well corresponding to direct contact with D145. Following D145 contact, the A211-H217 loop was able to relax around Cd(II) to its original conformation (Fig. 6e). The resulting hUNG structure with Cd(II) bound at the active site closely resembled the functional protein conformation, e.g. when the catalytic water is present. The patterns in PC1 displacement data agreed with the energy barriers from SMD simulations and indicated that the major energy barriers could be due to unfavorable strain on hUNG during maximal expansion/contraction of the DNA binding groove.

## Discussion

To our knowledge this is the first study focusing on metal inhibition of hUNG. As previously described^18^ the key issue in these studies is to assess metal-induced effects at their environmentally and biologically relevant concentrations. The concentrations of Cd(II) in organs/tissues of healthy unexposed humans can be in the low to middle micromolar ranges. Studies showed that the levels of Cd(II) reach 12–28 μM in the prostate, 0.9–6 μM in lungs, and significantly higher levels in kidneys and liver^15^,^28^,^29^. Under certain circumstances, levels of Cd(II) could be significantly higher in cells, reaching 100 uM ranges. Normally most cellular Cd(II) is bound to an intracellular protein called metallothionein^30^, however under oxidative stress Cd(II) may be released from metallothionein leading to higher levels in cells. Moreover, cadmium has a fairly long biological half-life (10–30 years), thus resulting in age-dependent accumulation in organisms. In this work, the inhibition of hUNG activity was observed at concentrations starting from 50 μM which should be considered biologically relevant.

The water activating 145-DPYH-148 loop of hUNG is needed to cleave the glycosidic bond between the lesion and DNA backbone using an activated water molecule. From examination of various crystal structures of UNG, the catalytic water was present regardless of whether DNA was bound (PDB:1SSP)^31^ or unbound (PDB:1AKZ)^31^ and also in the case of a bound inhibitor (PDB:2J8X)^32^. The catalytic water was observed in all examined crystal structures suggesting that the water activating loop provides a conserved site for the catalytic water. Recent computational work showed that H148 can assume the role of water activation in the catalytic mechanism and additionally showed that D145 was required to stabilize the transition state^33^. Oxygen donor ligands are known to have synergistic effects with imidazole groups when binding transition metals^34^. The active site of hUNG possesses the oxygen donor and imidazole group motif via the D145/H148 residues, supporting our findings that Cd(II) ions inhibit hUNG by displacing the catalytic water and thereby preventing base excision. Additionally, the DNA binding groove of hUNG is known to be positively charged to facilitate DNA binding which was shown computationally via electrostatic calculations^35^. This characteristic of UNG should present a challenge for positively charged metal ions to contact the water activating loop which is enveloped by the positive charge of the DNA binding groove. However in our work we showed this energy barrier was relatively small (12 kcal/mol) and under physiological temperatures can be breached^36^.

Finally we compared our proposed binding site against the MetalPDB database^37^ of metal containing crystal structures to determine if it was consistent with empirical structural data. Our search revealed that our proposed site exactly matched 10 crystallized Cd(II) binding sites utilizing a carboxyl group (aspartate or glutamate), imidazole group (histidine), and a peptide oxygen. We then relaxed the search to include exactly one aspartate/glutamate carboxyl and one histidine imidazole which we considered a “UNG-like” binding site and known to be a favorable motif for metals^34^. This search revealed 210 sites, or 6.8% of all cadmium sites. Compared to other metals tested in HeLa cell extracts in this work, Zn(II) matched 5.3% (1092), Ni(II) matched 5.6% (89), and Pb(II) matched none likely due to a limited amount of data (*N* = 138). This search showed that Cd(II) favored UNG-like sites more than both Zn(II) and Ni(II) which supported the biochemical data in this work.

Extending our findings to other DNA repair enzymes offered valuable insight. Our previous work with MPG showed that Zn(II) inhibited activity in the 1000 μM range and was due to the binding a glutamate carboxyl, tyrosine hydroxyl, and an amide oxygen^18^. Based on our current work, it is possible to propose that weaker binding to this site was due to the absence of a supporting histidine imidazole since metal ions inhibited MPG approximately 10 times weaker than UNG. APE1, an endonuclease that requires metal for activity, was inhibited by Cd(II) ions in the 10 uM range, or roughly 5 times stronger than UNG^13^. APE1 binds catalytic Mg(II) with two carboxyl groups (D70:E96), but interestingly also has an adjacent UNG-like binding site where H309 is surrounded by 4 glutamate or aspartate residues which includes the D70:E96 binding site. Based on existing structural data (PDB ID 4QHE), it is likely that metal ions such as Cd(II) could bind tightly to the multiple residues and explain why Cd(II) was able to bind APE1 stronger than UNG. Other glycosylases also show similar metal binding motifs, which make them possible targets for toxic metal inhibition. For example, single-strand selective UNG (SMUG1) excises uracil primarily from single stranded DNA and has a single UNG-like binding site (D146:H250; PDB ID 1OE4) adjacent to bound DNA. Another BER glycosylase, thymine-DNA glycosylase (TDG), contains several UNG-like sites as well as an APE1-like motif (D126:E194; PDB ID 4JGC). However, there is currently no known data regarding metal inhibition for these glycosylases.

In summary, the biochemical data presented in this work was a first look at hUNG inhibition by metal ions. Our *in vitro* biochemical data confirmed that Cd(II) can inhibit the catalytic activity of human UNG at micromolar concentrations. Inhibition of UNG activity prevents repair of DNA damage which could lead to mutagenic and carcinogenic events in cells. The computations in this work provided a landscape to how Cd(II) interacts with hUNG, a non-metal requiring enzyme, and further showed that the modeled interactions can ultimately lead to inhibition by active site binding. The presence of the identified UNG-like metal binding motif in other DNA repair enzymes could indicate toxicity from metals such as Cd(II).

## Methods

Caution: Cadmium, nickel, and lead compounds are classified carcinogens and should be handled with appropriate protection in accordance with NIH guidelines.

### Chemicals, proteins, and synthetic oligonucleotides

Cadmium chloride (CdCl_2_), nickel chloride (NiCl_2_), zinc chloride (ZnCl_2_), and lead chloride (PbCl_2_) were purchased from Aldrich-Sigma (St. Louis, MO). Stock solutions at 0.1 mM concentrations were prepared using distilled and deionized water obtained from the Millipore ultrapurification system. Cell-free extracts from HeLa S3 cells (Cell culture Center, Endotronics, Minneapolis, MN) were prepared as described previously^38^. Truncated human recombinant protein hUNG was obtained from Enzymax (Lexington, KY). The 25-mer oligonucleotide containing a single U at the 6th position from the 5′ side (5′-CCGCTUGCGGGTACCGAGCTCGAAT) was synthesized as described previously^39^. The complementary strand and 7-mer size markers were synthesized either with an Applied Biosystems Model 394 automated DNA synthesizer or purchased from Operon (Louisville, KY). All modified and unmodified oligomers were HPLC- and/or PAGE-purified.

### DNA glycosylase assay

To prepare the radiolabeled DNA substrate, the U-containing 25-mer oligonucleotide was 5′-end labeled with [μ-32P] ATP (specific activity 6,000 Ci/mmol; 1 Ci = 37 GBq, Amersham Pharmacia Biotech, Piscataway, NJ) and T4 polynucleotide kinase (United States Biochemical, Cleveland, OH) according to standard procedure. The ^32^P-labeled oligomer was subsequently annealed to a complementary strand in a molar ratio of 1:1.5. A DNA cleavage assay was carried out as described previously^21^,^38^. The reaction mixtures contained 2 nM ^32^P-end labeled oligomer duplex in 10 mM HEPES-KOH (pH 7.4), 100 mM KCl, 0.1 mM DTT, and varying amounts of purified hUNG protein in a total volume of 10 μL. Metal ions were pre-incubated with hUNG for 10 min on ice prior to the addition of the substrate DNA. Further incubations were carried out for 30 min at 37 °C. In reactions using cell-free extracts, 0.5 μg of poly(dI-dC)-poly(dI-dC) (Amersham Pharmacia Biotech, Piscataway, NJ) was added to the reaction mixture as a nonspecific competitor. All glycosylase reactions were stopped by adding 5 μL alkaline buffer (300 mM NaOH, 90% formamide) followed by heating the samples at 95–100 °C for 3 min. Reaction products were resolved on a 7 M urea 12% denaturing PAGE with a 5′ ^32^P-labeled 5-mer marker. The gel was subsequently dried and scanned with the Bio-Rad FX Molecular PhosphorImager (Hercules, CA). For band quantitation, Quantity One software (version 4.0.1) was used according to the manufacturer’s instructions.

### Molecular dynamics

Molecular dynamics (MD) simulations were calculated using the AMBER 12 package^40^. The PDB ID 1AKZ was solvated with TIP3 water in a periodic box with at least 12 Å of water on all sides of the protein, giving dimensions of approximately 79 Å × 70 Å × 77 Å. The ff99SB force field^41^ was used for the MD parameters. The timescale used in the biochemical experiments for the protein/Cd(II) incubation cannot be reproduced by current MD simulation procedures. Moreover, a simulation corresponding to a 100 μM Cd(II) concentration would require a water box 3 times larger resulting in about 32 times more water molecules, causing the simulations to become prohibitively slow even at shorter timescales. We therefore increased the Cd(II) concentration compared to our assays using 50 ions. The high number of ions ensured accelerated sampling of Cd(II) around the entire surface of the hUNG at the μs timescale. Parameters used for Cd(II) were taken from the work of de Araujo and coworkers, which reported 2.7 Å and 0.025 kcal/mol for the VDW diameter and well-depth, respectively^42^.

The systems were of the NPT ensemble using the Langevin thermostat and a weakly coupled barostat. The time step was 2 fs using SHAKE to freeze bonds to hydrogen, and a 12 Å cutoff was used for short range electrostatics with long range electrostatics using the Ewald summation method^43^. The particle mesh ewald molecular dynamics (pmemd) program using GPU acceleration^44^ was used to run the simulation with all other parameters at their default value as set in AMBER 12.

Each simulation with and without Cd(II) was prepared using two minimization stages, one heating stage, and two equilibration stages. The minimization procedure used 500 steps of steepest descent followed by 500 steps of conjugate gradient with a 10 kcal/mol harmonic restraint. This was followed by a minimization with the restraint removed. The system was then heated from 100 K to 300 K over a period of 50 ps and then allowed to adjust to proper density by first restraining the protein with 10 kcal/mol for 500 ps and followed by another 500 ps with no restraint. The production was run for a total 1 μs for each system. The radial distribution function of Cd(II) ions around the active site were constructed using a 0.25 Å spacing. Principal component analysis (PCA) was performed using the functions provided by cpptraj in the AMBER package. The 645 × 645 covariance matrix used for PCA was calculated from the alpha carbons of the inner 215 residues, omitting the first and last 4 residues, of the 50 Cd(II) simulation. The displacements, or projections, of the PCs were calculated as the inner product between the snapshot in the MD trajectory and PC, defined mathematically as 〈*x*_*i*_, *e*_*j*_〉 for the 1 × 645 row vector snapshot 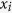 and 645 × 1 column vector PC*j*. The trajectories from the Cd(II) and metal-free simulations were projected onto the first PC (PC1) of the PCA, producing a distribution of displacements for each trajectory.

Steered MD (SMD) simulations were performed using methods of our previous work^45^. In order to capture both the long and short range behavior of UNG in response to the pulled Cd(II) ion we increase the water box to 15 Å from 12 Å, giving dimensions of approximately 85 Å × 76 Å × 83 Å. A Cd(II) ion was placed 32.2 Å away from catalytic D145 based on the PDB structure and pulled 30 Å at 0.075 Å/ns (400 ns). This procedure was repeated 4 times resulting in 1.6 μs total simulation time. A force of 20 kcal/mol/Å was used as the guiding potential modeling the “stiff spring” approximation^46^. As a result Cd(II) closely followed the reaction coordinate and the slow pulling of 0.075 Å/ns minimized error by maintaining reversibility of the process. From a protein unfolding experiment using the same approach, a pulling speed of 0.1 Å/ns was found to be reversible^47^. This class of PMF using SMD is based on the Jarzynski equality^48^ and is implemented in AMBER.

QM/MM MD was configured using the PM3 semi-empirical model in the sander program of AMBER, using equivalent settings for the MM region described above. The PM3 region included Cd(II) and the sidechains of D145, P146, H148, and H268. For this simulation, the time step was reduced from 2 to 1 fs and SHAKE turned off for the QM region. This system was run for a total of 8.5 ns using an 8 Å cutoff for QM/MM electrostatics. The coordinates produced by the QM/MM MD simulation were used as the initial structure for the DFT geometry refinement.

### DFT geometry refinement

We utilized the ONIOM method of Gaussian09 (Gaussian, Inc., Wallingford CT, 2009) which has already been successfully used to study DNA repair mechanisms^33^,^49^,^50^,^51^. We used the LC-ωPBE/AMBER hybrid potential with the 6-311+G(d) basis set for C, N, O, and H atoms and the def2-TZVPD basis set^52^ for the Cd(II) ion which described the 20 valence electrons with a triple-ζ basis set with polar and diffuse functions, while the first 28 electrons were described by the def2 effective core potential (ECP)^53^.

The starting structure for refinement was taken from the QM/MM MD simulation. The structure was first minimized with sander of AMBER using the same QM/MM settings used to produce the initial structure. The system first underwent 500 steps of steepest descent followed by conjugate gradient minimization, which was considered complete when the energy gradient was less than 0.0025 kcal/mol (on the order of 10,000 steps). This extra minimization step greatly helped the convergence of the DFT calculations. The LC-ωPBE layer consisted of Cd(II), one water molecule, the sidechains of catalytic D145 and H148, the local peptide oxygen of P146, and adjacent hydrogen atoms to satisfy link atom rules. The convergence criterion was set to “normal”, and a frequency analysis followed which reported no imaginary frequencies.

### DFT considerations

DFT for accurately handling transition metals is still an active area of research as the exact electron-exchange functional is not known. Functionals are regularly parameterized in order to satisfy the element groups they are designed for. Functionals which may perform well for organic chemistry may perform poorly for inorganic chemistry^54^. For example, the popular hybrid DFT functional B3LYP, which has a good combination of speed and accuracy, has been shown to be of poor quality for transition metal complexes^55^,^56^. Functionals which are local, i.e. have no HF electron-exchange, are good for modeling transition metals but perform worse than others for main group chemistry^57^. Thus it is difficult to know which DFT method will provide reliable results for a metal ion interacting with a non-metal requiring enzyme such as UNG. Indeed, we initially tested a variety of functionals and found the results varied considerably. The M06-2X functional has been shown to model the catalytic step in hUNG and other glycosylases with good accuracy^33^,^49^,^58^, but these reports did not include any metal. The M06-2X functional uses a high HF exchange coefficient and describes main group chemistry better than its M06-L counterpart^57^, however high HF exchange is known to be unfavorable for transition metals. The M06-L functional removes exchange in effort to describe open-shelled transition metals more accurately in addition to being parameterized specifically for transition metals. We study the closed shell Cd(II) cation in this work which removes the issue of multireference inherent in transition metals, a known difficulty for DFT. Given the fact that Cd(II) has a complete d-shell, multireference character should be limited and thus we would not enjoy the main benefit of the M06-L functional sought to provide^59^,^60^,^61^. The PBE-based functional has been shown to a good choice in a variety of chemical environments including those involving metal centers^62^. We chose the long-range corrected LC-wPBE functional since it appeared well-rounded^63^ and long range dispersion effects were found to be required for accurate modeling of transition metals^64^,^65^. When dispersion corrections are ignored bond lengths tend to be overestimated^65^.

Effective core potentials (ECPs) have been successful in modeling transition metals with negligible accuracy penalties combined with large computational efficiency gains^65^,^66^. ECPs, such as those of the Stuttgart-Dresden (SD) type, utilize a scalar relativistic correction which is important for second row transition metals and below^53^. This relativistic correction is ignored in all but the more expensive functionals, promoting the use of ECPs with DFT functionals. The 6-31+G(p) basis set utilizing SD ECPs has been shown to be in similar in accuracy to using the quadruple-ζ quality QZVP basis set for second row transition metals^67^.

## Additional Information

**How to cite this article:** Gokey, T. *et al*. Cadmium(II) inhibition of human uracil-DNA glycosylase by catalytic water supplantation. *Sci. Rep.* **6**, 39137; doi: 10.1038/srep39137 (2016).

**Publisher's note:** Springer Nature remains neutral with regard to jurisdictional claims in published maps and institutional affiliations.
